# Urinary neopterin, a non-invasive marker of mammalian cellular immune activation, is highly stable under field conditions

**DOI:** 10.1038/srep16308

**Published:** 2015-11-09

**Authors:** Michael Heistermann, James P. Higham

**Affiliations:** 1 Endocrinology Laboratory, German Primate Centre, Leibniz Institute for Primate Research, Goettingen, Germany; 2 Department of Anthropology, New York University, New York, NY, USA

## Abstract

Studying immunity and immune function in ecology and evolution requires field studies, but there has been a dearth of non-invasive markers of immune activation available for studying large wild mammals. Recently, we analytically and biologically validated the measurement of urinary neopterin (NEO), a biomarker of cellular immune activation, in captive macaques. However, applying this to free-ranging settings is complicated by issues involving sample collection, processing, storage, and transport. Here, we collected urine samples from captive macaques and undertook experiments simulating common field issues. We tested the effects on urinary NEO sample measurements following: dirt and faecal contamination; storage at room temperature; differences in processing and long-term storage methods (freezing, lyophilising, blotting onto filter paper); and freeze-thaw cycles. Our results show that concentrations of urinary NEO are highly stable – they are not affected by soil or faecal contamination, can be collected on filter paper and stored for many months frozen or lyophilised with minimal effect, and are resistant to multiple 24 hr freeze-thaws. With the addition of a biocidal preservative, concentrations are even stable at room temperature for long periods. Urinary NEO is remarkably resilient, and is highly suitable for non-invasive field studies of cellular immune responses in wild large mammals.

The immune system, which acts to protect the body from disease, is a critical element of animal biology, and is therefore key to understanding the ecology and evolution of animal species. Immune system effectiveness and responsiveness are important variables for anticipating the likely spread of infections through populations, and for an understanding of disease ecology, and population and conservation biology^1^,^2^,^3^. Outside of ecology, there are also many areas in evolutionary biology that include the immune system as a critical element. These include theories such as the immuno-handicap hypothesis^4^,^5^, MHC complementarity in mate choice^6^, and theories on the evolution of immunity^7^.

For a full understanding of the role of physiology and immunology in animal ecology and evolution, studies in natural settings are clearly needed. For some terrestrial taxa, natural settings do not prohibit the collection of blood. For example, birds can be mist-netted for blood collection, while small mammals and reptiles can be trapped, and re-released. For large mammals, such methods are rarely an easy option. Instead, for mammalian studies, there has been a methodological focus on the non-invasive measurement of physiology through the collection of excreta^8^. This has been transformative for studies of the behaviour and life-history of large free-ranging mammals. For research on nonhuman primates, early methodological progress came in the form of the measurement of female reproductive status through progestogen and estrogen values measured from urine^9^,^10^, while the late 1980s and early 90s saw the first studies utilizing faecal measurements of these parameters^11^,^12^. Since then, other developments in steroid hormone analysis have included the measurement of faecal and urinary glucocorticoids and androgens^13^,^14^,^15^,^16^. More recently still, studies have begun to diversify further, incorporating thyroid hormones measured from urine^17^ and faeces^18^ and measurements of urinary proteins and peptides, such as concentrations of urinary C-peptide of insulin^19^,^20^,^21^,^22^ and oxytocin^23^.

Despite great strides in such physiological assessment, the validation and implementation of non-invasive measures of immune function and immune response activation have been lacking. Primate health and condition has usually been assessed in the field indirectly by visual estimates of wounding^24^ and levels of body fat^25^, simple dipstick urinalyses^26^,^27^ or by the collection of stool samples for parasitological analysis^28^. In a recent publication, we evaluated the potential utility of potentially informative non-invasive markers for monitoring immune activation and infection in macaques^29^. We assessed three biomarkers of potential interest -the acute phase proteins C-reactive protein and haptoglobin, and urinary NEO, which is produced by monocytes/macrophages upon stimulation with interferon gamma (IFNγ), interferon alpha (IFNα), other cytokines, and endotoxins. Measurement of urinary NEO concentrations in body fluids like serum or urine provides information about T helper cell 1 derived cellular immune activation^30^,^31^. Our results for urinary NEO were particularly promising. First, urinary NEO concentrations were strongly and highly significantly correlated with those in serum. Second, they showed a strong and highly consistent response to viral infection, increasing in concentration 10–25 fold in response to inoculation with SIV^29^. Indeed, this latter result is well known within the pathology literature, where concentrations of urinary NEO are regularly assayed to evaluate successful infection of macaques with SIV^32^. In humans, increased urinary NEO production (and excretion) is also found in infections by intracellular living bacteria and parasites, and in malignant tumor diseases^31^. It has also been implicated with intestinal infections, inflammation and macrophage activity^33^,^34^, as well as with oxidative stress^31^, indicating its potential as a general biomarker of health and disease status.

Despite the promise of urinary NEO as a non-invasive measure of immune activation, a lot of issues remain before it might usefully be applied to field studies, especially those operating in remote and challenging natural environments. One potential issue is already known—neopterin is sensitive to direct sunlight (though not light more generally), such that samples should be shielded from direct sunlight during collection and then placed in the dark^35^. However, there are many other potential field issues that have not been tested. These include: regular cross-contamination of faecal and urine samples when either sample type is collected in the field, and contamination of both matrices with dirt; storage issues, such as how to stabilize samples before they can be returned to a field station or freezer, and how to store samples in the field (e.g. on filter paper, by freezing); how samples might be transported and the effects of potential freeze-thaws on analyte concentrations; and the question of whether and how samples might be stored long-term (e.g. for months) without degrading before they can be analysed in a laboratory. These types of problems have been addressed several times for the non-invasive measurement of steroid hormones^36^,^37^,^38^,^39^, and also for urinary C-peptide^22^.

Here, we make similar assessments for urinary NEO, as an attempt to evaluate its potential utility for field studies of mammalian cellular immune activation. We do this by testing different treatments of urine subsamples against control subsamples taken from the same urine void. First, we tested for potential effects of soil and faecal contamination upon collection by adding these contaminants to urine samples. Although neopterin is not present in large concentrations in faeces, and so risks of cross-contamination are not great, both soil and faecal matter may potentially absorb neopterin so reducing urinary concentrations, while faecal enzymes may also degrade it. Next, we addressed issues of short- to medium-term processing and storage. Under remote field conditions, the opportunity to freeze urine samples within a short-time window (e.g. a couple of hours) upon collection may not always be possible due to the impracticality of running a freezer at a field site without power. It is sometimes possible, however, to operate a freezer in a nearby village where samples can regularly be brought for freezing. Since this, however, may be impractical on a daily basis, samples might potentially have to be stored unfrozen for periods from a couple of days to a few weeks before being placed in a freezer. Similarly, it may be difficult to keep urine samples in a frozen state during transportation to the laboratory where analyses are undertaken. Under such circumstances, urinary analytes may degrade due to microbe growth and microbial activity^22^,^36^. We therefore tested for degradation of urinary NEO concentrations when samples were stored unpreserved at room temperature for periods of up to three weeks and with the addition of a biocide preservative (ProClin 200), for storage durations of up to four months. We also tested the effects of processing of samples and subsequent re-suspension following two commonly used storage procedures—blotting onto filter paper and lyophilisation. We then assessed issues of long-term storage and shipment, including the effects of being stored for months frozen, and the effect of being thawed and kept at 24 hours at room temperature, a treatment that might occur during laboratory analyses, or during a power cut in the field, but also potentially during transport if frozen samples cannot be shipped quickly and/or with sufficient ice or dry ice. As most researchers are likely to index their measured urinary NEO concentrations by assessing creatinine (Cr) concentrations, and as Cr field robustness is a question of interest in its own right, we first present results for urinary Cr in response to all treatments. We then present urinary NEO concentrations indexed for Cr, as this indexed measure is the one that most researchers would calculate and present in publications. Our overall aim was to evaluate the likely utility of urinary NEO for field studies that wish to incorporate measures of cellular immune responses. As to our knowledge there have been no prior tests of such conditions on urinary NEO concentrations, we made no specific predictions about the effects that such treatments might have.

## Results

### Creatinine measurements

Generally, Cr concentrations showed no or only weak effects in response to the different experimental treatments. They were not statistically affected by contamination with soil (T^+^ = 18, N = 12, P = 0.197), but showed a small and significant decline in concentrations to 95.7 ± 1.2% (mean ± s.e.m.) of control values in samples contaminated with faeces (T^+^ = 3, N = 12, P = 0.020). However, such a small decline seems unlikely to have major effects on results as it is within typical measurement variation and error (within assay variability). Although Cr concentrations were apparently affected in samples containing ProClin 200 as preservative when stored at room temperature for up to 3 weeks (χ^2^ = 8.296, df = 3, P = 0.04), in fact the strongest decline in concentrations recorded was to 97.1 ± 1.2% of controls (day 10). Post-hoc statistical comparisons of the separate storage days to controls did not reveal a statistically significant effect of any day (all P > 0.15), suggesting that the overall statistical result is likely to be an anomaly. The only treatments that caused more substantial changes in Cr concentrations were the use of filter paper, the storing of samples without a preservative at room temperature for up to 21 days, and the long-term storage of ProClin-preserved samples at room temperature for 2 and 4 months. Blotting onto filter paper and subsequent reconstitution caused a decline in concentrations to 84.3 ± 2.4% of controls (T^+^ = 0, N = 12, P < 0.001); storage of samples at room temperature for three weeks resulted in a decrease in concentrations to 72.6 ± 4.8% of controls (χ^2^ = 32.00,df = 4, P < 0.001), with the first significant decline being noticed at day 7 of the treatment period (T^+^ = 0, N = 12, P < 0.001). Storage of preserved samples for 2 and 4 months caused a decline in Cr concentrations to 87.7 ± 4.0% and 80.4 ± 3.6% of controls, respectively (χ^2^ = 22.800, df = 2, P < 0.001), with changes at both time points being significant (each T^+^ = 0 N = 12, P < 0.001). Nevertheless, results following all experimental treatments remained strongly and significantly correlated with control values (all r_s_ > 0.92, all P < 0.001).

### Neopterin Measurements

Table 1 presents a summary of the urinary NEO results indexed for Cr, which are considered here in more detail.

### Collection And Storage Issues

#### Contamination With Soil And Faeces

**Figure 1:**
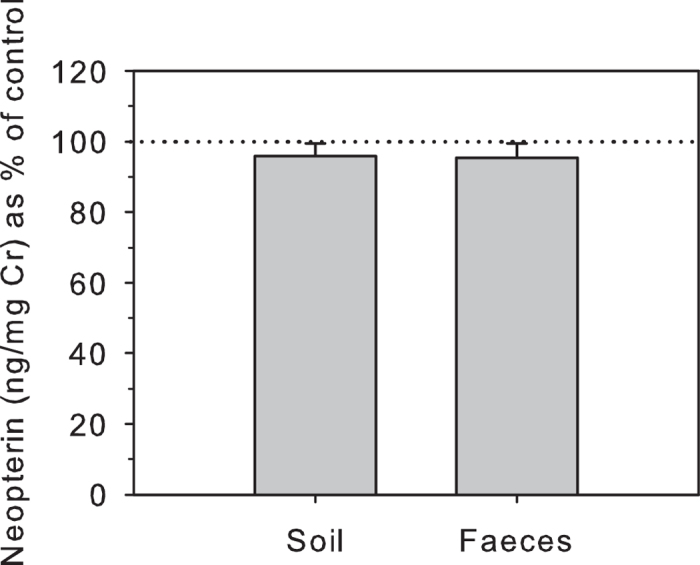
Effect of soil and faecal contamination of urine samples on urinary NEO (n = 12). Urinary NEO concentrations are shown as percentage (mean** ± **s.e.m.) of control values (100% shown by dotted line).

Soil and faecal contamination did not have significant effects on urinary NEO concentrations. Samples contaminated with soil declined in urinary NEO concentrations to 96.1 ± 3.6% (mean ± SEM) of controls (Fig. 1), which did not represent a statistically significant reduction (T^+^ = 18, n = 12, P = 0.206). The same applied to samples contaminated with faecal matter, where urinary NEO concentrations declined to an average 98.3 ± 4.2% of controls (Fig. 1), again a non-significant decline (T^+^ = 22, n = 12, P = 0.204). Urinary NEO concentrations were strongly and highly significantly correlated with controls following both treatments (both r_s _> 0.93, n = 12, P < 0.001).

#### Storage Of Samples At Room Temperature For Various Periods With And Without A Preservative

**Figure 2:**
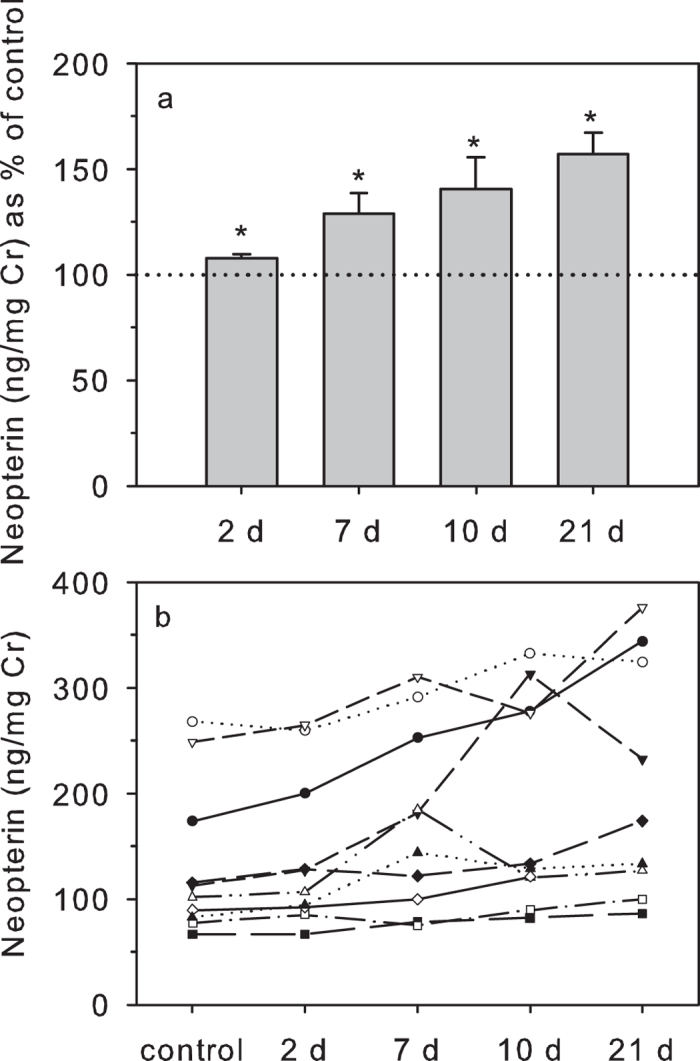
Effects of room-temperature storage on urinary NEO (for up to 3 weeks). Urinary NEO concentrations (n = 11) are shown as: (**a**) percentage (mean ± s.e.m.) of control values (100% dotted line); and (**b**) absolute concentrations. For each treatment, *indicates concentrations that differ significantly from controls. d = days of storage.

Storing urine samples unpreserved at room temperature for up to 21 days resulted in gradually increasing rises in urinary NEO concentrations over the experimental period compared to controls (2 days: 107.6 ± 2.0%; 7 days: 129.0 ± 9.1%; 10 days: 140.4 ± 14.4%; 21 days: 157.1 ± 9.3%; Fig. 2A). These increases were highly significant over the whole period and represented rises above 100% of controls (χ^2^ = 27.839, df = 4, P < 0.001). Post-hoc Wilcoxon tests showed that the rise in urinary NEO concentrations was already statistically significant at Day 2 of storage (T^+^ = 50, n = 11, P = 0.020), although the magnitude of the increase was small and within assay variability. Moreover, the rank order of the different individual samples (from high to low) at this day of storage remained largely unchanged (Fig. 2B), and concentrations at Day 2 very strongly correlated with those of controls (r_s_ = 0.973, n = 12, P < 0.001). Urinary NEO concentrations were also significantly higher at Day 7 (T^+^ = 65, n = 11, P = 0.002), Day 10 (T^+^ = 66, n = 11, P = 0.001) and Day 21 (T^+^ = 55, n = 11, P = 0.002). Increases in urinary NEO values at these days were sufficiently variable between samples to change their relative rank order more markedly (Fig. 2B). Concentrations on these days were nevertheless still significantly (but less strongly) correlated with controls (r_s_ value range: 0.845 – 0.891, n = 11; all P < 0.003).

A look at the raw data revealed that the rise in Cr-indexed NEO concentrations during the treatment period was apparently due to the concomitant decrease in Cr concentrations (see above). Indeed, urinary NEO levels expressed per volume of urine (i.e. per mL) rather than per mass Cr did not change significantly over the treatment period (χ^2^ = 3.0802, df = 4, P = 0.5445), indicating that, in contrast to Cr, urinary NEO itself was unaffected by the treatment.

**Figure 3:**
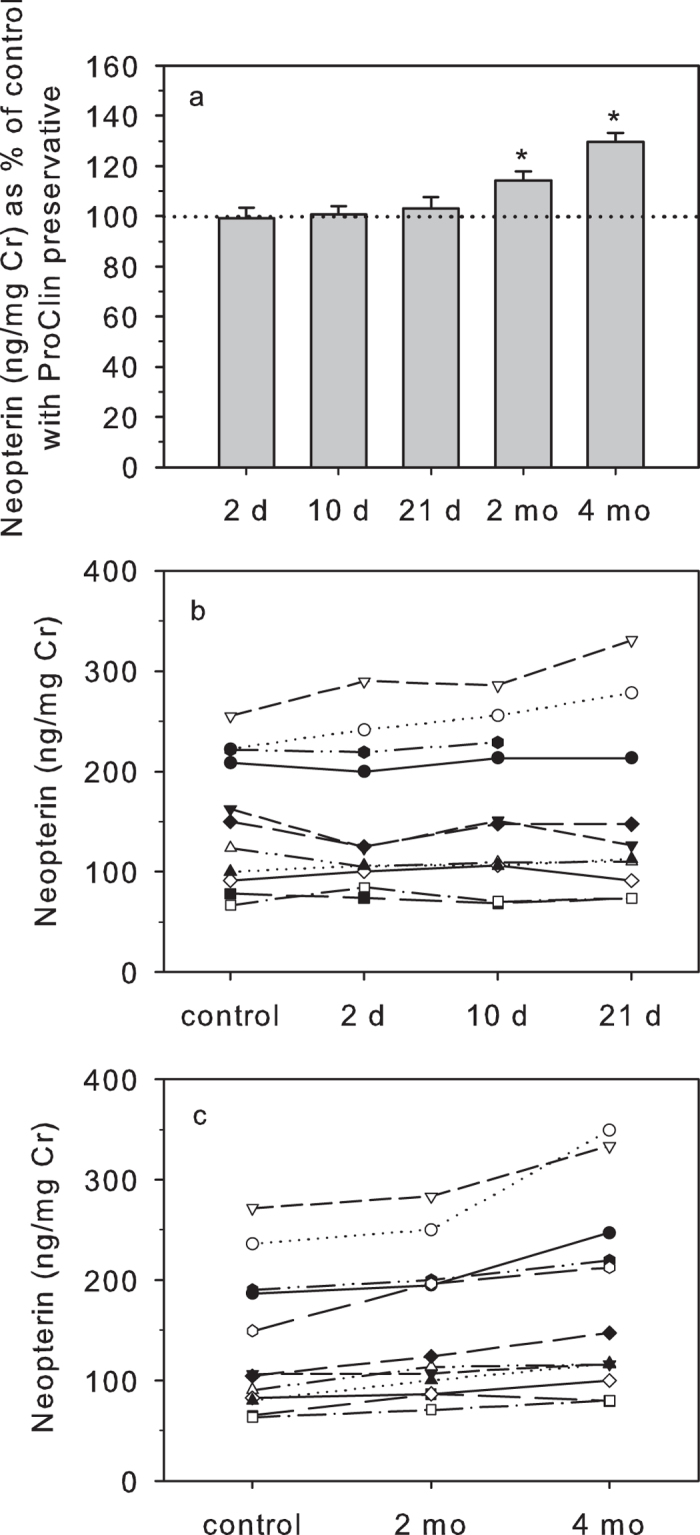
Urinary NEO concentrations in samples containing ProClin 200 as a preservative and stored at room temperature for up to 4 months. Urinary NEO concentrations are shown as: (**a**) percentage (mean ± s.e.m.) of control values (100% dotted line); and (**b,c**) absolute concentrations. For each treatment, *indicates concentrations that differ significantly from controls.

Adding ProClin 200 as a preservative to the urine samples prevented the alterations in urinary NEO concentrations seen in unpreserved samples over the 21 day storage period (χ^2^ = 1.404; df = 3, P = 0.705). Urinary NEO concentrations in urine containing the biocide were similar to those measured in controls at all days of the experimental period (Day 2: 99.2 ± 4.1%; Day 10: 100.7 ± 3.2%; Day 21: 103.0 ± 4.6%; Fig. 3A). Also, and in contrast to the situation in unpreserved urine, the rank order of the samples remained basically unchanged (Fig. 3B), and at each time point of the experimental period urinary NEO concentrations very strongly correlated with those of controls (all r_s_ > 0.96, n = 11, all P < 0.001).

Longer-term storage of preserved urine for up to 4 months resulted in a gradual and statistically significant increase in urinary NEO concentrations compared to controls (χ^2^= = 22.1667; df = 2, P < 0.0001); Fig. 3A). Post-hoc Wilcoxon tests showed that the rise in urinary NEO concentrations was already statistically significant after 2 months of storage (T^+^ = 78, n = 12, P < 0.001). However, the magnitude of this rise was relatively small (14.3 ± 3.4%), the rank order of the individual samples remained largely the same (Fig. 3C), and concentrations very strongly correlated with those of controls (r_s_ = 0.965, n = 12, P < 0.001). In contrast, urinary NEO concentrations showed a more marked elevation at 4 months (29.7 ± 3.6% rise; T^+^ = 78, n = 12, P < 0.001) and rank order of samples was more disturbed (Fig. 3C). Concentrations were nevertheless still strongly correlated with controls (r_s_ = 0.923, n = 12, P < 0.001). Urinary NEO concentrations expressed per volume of urine did not change significantly over the treatment period (χ^2^ = 0.650, df = 2, P = 0.7225), indicating that, similar to unpreserved samples (see above), the rise in Cr-indexed urinary NEO concentrations was due to the storage-induced decrease in Cr concentrations (see above).

#### Collection Of Samples Onto Filter Paper

**Figure 4:**
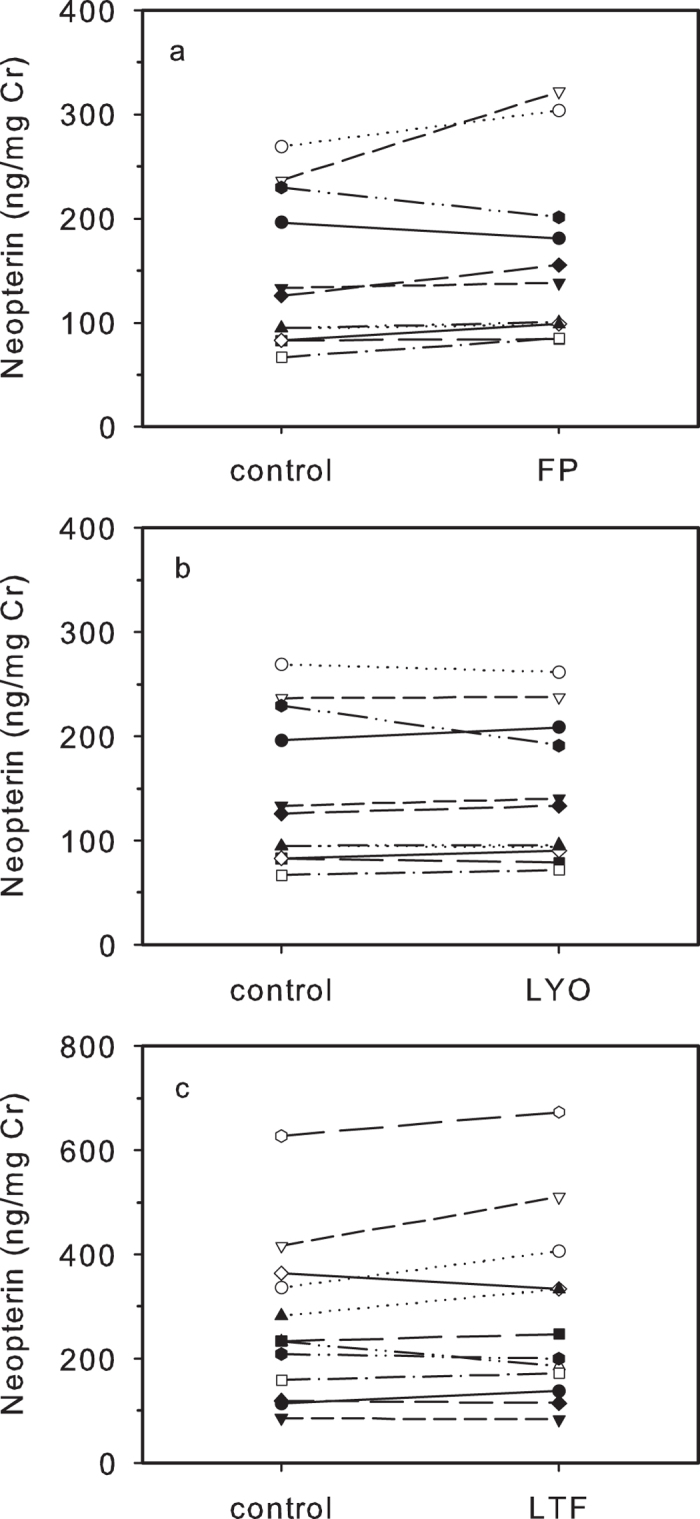
Effect of filter paper collection FP (a), lyophilisation LYO (b), and long-term freezing LTF (c), on urinary NEO concentrations (all n = 12).

Samples blotted onto filter paper and reconstituted two days later had higher NEO concentrations when compared to controls (110.4 ± 4.1%). However, the magnitude of this elevation was small and non-significant (T^+^ = 64, n = 12, P = 0.052), the rank order of samples remained largely unchanged compared to controls (Fig. 4A), and urinary NEO concentrations were strongly and significantly correlated with controls (r_s_ = 0.951, n = 12, P < 0.0001).

### Processing And Long-Term Storage

#### Lyophilisation

Lyophilized samples showed no significant change in urinary NEO values compared with controls (100.6 ± 2.0%; T^+^ = 44, n = 12, P = 0.718), and due to very low variation in changes between sample concentrations as a result of this process, the rank order of the samples changed little (Fig. 4B). Urinary NEO concentrations also very strongly correlated with those of controls (r_s_ = 0.993, n = 12, P < 0.001).

#### Long-term Freezing

Urinary NEO concentrations in samples stored for 8 months frozen increased to 105.4 ± 3.9% of controls, a statistically non-significant increase (T^+^ = 27, n = 12, P = 1.0). Moreover, the rank order of the samples changed little (Fig. 4C) and urinary NEO concentrations were very strongly correlated with those of controls (r_s_ = 0.974, n = 12, P < 0.001).

#### 24-hour Freeze-Thaw Cycle

**Figure 5:**
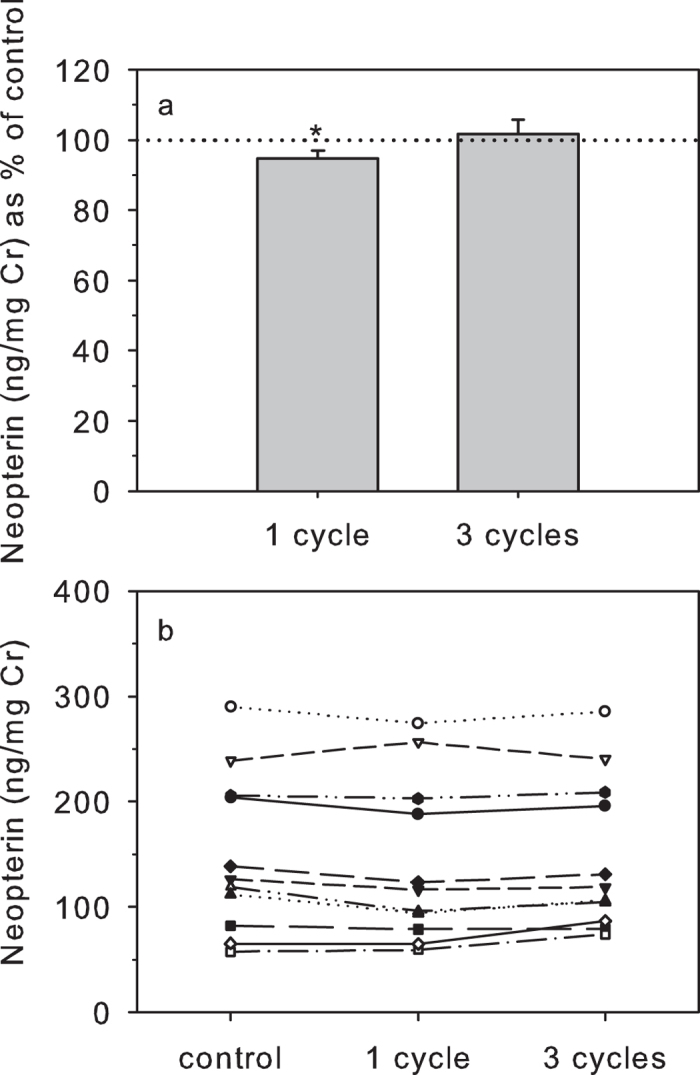
Effect of exposing urine samples to one and three 24 h freeze/thaw cycles on urinary NEO. Urinary NEO concentrations are shown as: (**a**) percentage (mean ± s.e.m.) of control values (100% dotted line); and (**b**) absolute concentrations. For each treatment, *indicates concentrations that differ significantly from controls.

Urinary NEO concentrations were affected by freeze-thaw situations (χ^2^ = 8.667; df = 2, P = 0.013). However, this effect was overall small (one freeze-thaw cycle: 94.8 ± 2.6% of controls; three freeze-thaw cycles: 101.6 ± 4.1% of controls; Fig. 5A) and within the range of within-assay variability. Moreover, a post-hoc Wilcoxon test revealed that only the change in urinary NEO concentrations following one thawing-refreezing process was significant (T^+^ = 13, n = 12, P = 0.042) while the change in urinary NEO concentrations recorded following three freeze-thaw cycles was not (T^+^ = 26, n = 12, P = 0.339). In addition, the rank order of samples remained basically unchanged after both one and three freeze-thaw cycles (Fig. 5B) and urinary NEO concentrations correlated very strongly to those of controls after both treatments (r_s_ > 0.98, n = 12, P < 0.001).

## Discussion

Our study aimed to test the likely utility of urinary NEO as a biomarker of cellular immune activation for use in wild and free-ranging settings, where there are many complications involved in sample collection, processing and storage. Specifically, our study served to characterize the stability of urinary NEO and to identify the conditions for optimal processing and storage of this immune marker under field-like conditions. We found urinary NEO to be remarkably resilient to many of the likely challenges that fieldworkers will face, and therefore conclude that it is likely to prove a highly practical marker for use in field studies. Although concentrations of urinary NEO itself were highly stable over time, rises in Cr indexed urinary NEO were detected due to degradation of Cr, indicating that it is mainly changes in Cr over time that researchers must consider when utilizing this marker. An alternative therefore, would be to consider indexing urinary NEO concentrations for specific gravity instead of Cr^40^, although we did not test this in the present study.

In our contamination tests, we found that urinary NEO is not sensitive to short periods of faecal or soil contamination, with both treatments having very slight and non-significant effects on its concentration. Nonetheless, as per with our treatment here, we recommend that samples are cleaned as soon as possible. This can be done in the field by allowing soil or faeces to settle to the bottom of the container into which urine is initially placed (for many species and studies a microcentrifuge tube), before transferring the supernatant into a clean tube^21^. Such cleaning is likely to be especially important if other markers, that are not as resilient to such contamination, are to be measured from the same sample (e.g. urinary C-peptide, which becomes highly degraded in the presence of faeces^22^).

Following this initial cleaning of samples, they can then either be kept at room temperature for up to 2 days with very minimal effect, or with the addition of a dilution of the biocide ProClin 200, for much longer. In our study we found no effects on urinary NEO concentrations of samples being kept at room temperature for up to 3 weeks once ProClin 200 had been added. This was due to a stabilizing effect on Cr which in unpreserved samples degraded gradually over time (as found elsewhere^22^,^36^,^37^) leading to a substantial increase in Cr-indexed urinary NEO concentrations. In samples stored longer-term (i.e. 2 and 4 months) however, the effectiveness of the preservative in stabilizing Cr was reduced, while absolute urinary NEO concentrations remained unaffected. Since the changes in concentrations of both Cr and Cr-indexed urinary NEO concentrations were nevertheless relatively minor after two months of storage (i.e. <15%), and as concentrations in samples stored for this duration correlated very strongly with controls, with no marked change in rank order between samples, we envisage that storing ProClin-treated samples for up to two months unprocessed should not affect results a great deal. Ideally, however, samples should be processed further (e.g. stored frozen or being lyophilized, see below) within a month of collection. If this is not possible, researchers should evaluate the effectiveness of higher concentrations of ProClin in the sample or that of other preservatives, such as ethanol, to stabilize Cr further^36^. In addition, researchers who plan to use their urine samples to measure other analytes should test the potential preservative effect of the biocide used on those analytes before adding it to their samples.

If samples are kept at room temperature for days or weeks, it is probably because they are being kept until they can be frozen or lyophilized. A potential alternative for situations where freezing samples is not possible at all would be to quickly blot cleaned samples onto filter paper^41^. Although this blotting and reconstitution had a small effect on indexed urinary NEO concentrations (due to incomplete recovery of Cr), it nonetheless still produced concentrations that were very strongly and significantly correlated with controls, and the overall change in concentrations was within typical measures of intra- and inter-assay variation. Thus collection onto filter paper seems potentially highly feasible for studies incorporating urinary NEO, although its stability on filter paper samples stored long-term needs to be evaluated.

Both lyophilisation and freezing seem highly suitable for long-term sample storage, with both processes having negligible effects on urinary NEO concentrations, similar to findings for other compounds, such as steroid hormones^38^ and C-peptide^22^. This is of course critical, as often samples cannot be analysed within short-time frames, and are typically stored at field sites for many months before shipment for analysis. In addition, multiple freeze-thaw cycles, which may occur under many circumstances, from losses of power at field stations and in remote rural tropical villages, to thawing during long transportation durations, also had minimal impact on urinary NEO concentrations. In sum, our multiple different treatments yielded only extremely minor effects on urinary NEO concentrations, suggesting that it is highly stable under field conditions.

Given that urinary NEO concentrations are likely to show specific elevations only upon infection, it will be necessary for field studies wishing to use this biomarker to collect regular samples from their study subjects. While laborious, this is not uncommon for such field studies. For example, for the non-invasive assessment of ovulation in female mammals, regular and frequent collection of samples is necessary for the measurement of estrogen and progestogen metabolites^43^. As such, regular urinary NEO sample collection might be pursued with similar methods and frequencies to those now commonly used for the measurement of ovulation^43^. Although some authors have suggested that urinary NEO is only produced in detectable forms and concentrations in humans and nonhuman primates^31^,^44^, a number of studies of stress and immune responses in other mammals have included measurements of serum NEO, suggesting the potential for much broader applicability (e.g. dogs^45^; pigs^46^; cattle, horses, lamas, dogs, cats and rats^47^).

In conclusion, urinary NEO seems to be a biomarker of high potential for application to field studies of wild and free-ranging mammalian populations. In macaques, concentrations of urinary NEO are strongly and highly significantly correlated with serum levels, and they show highly consistent and easily detectable responses to viral infection^29^,^32^. In addition, we have shown here that urinary NEO is highly stable and robust under the conditions in which it is likely to be collected, processed and stored in the field prior to analysis. It should be noted however that we undertook our testing using samples from captive macaques. Samples from wild macaques may differ in sample concentrations and pH in ways that impact these effects. Some testing of samples collected from the field is therefore recommended. More generally, the stability of urinary NEO in samples of other species may differ—for example, faecal contamination may lead to degradation of urinary NEO in samples from other species due to the presence of enzymes not present in the faeces of macaques. We therefore strongly encourage other researchers to undertake similar tests for their own study species.

There are a great number of topics where regular measurement of mammalian cellular immune activation might add substantially to the aims of the study. The ultimate test of the utility of urinary NEO will be in further studies which collect this biomarker in the field at the same time as other key variables, and investigate whether variation in urinary NEO concentrations is informative with respect to data on other aspects of animal ecology, physiology and behaviour.

## Materials and Methods

### Study Animals And Sample Collection

Urine samples were collected from twelve healthy adult rhesus macaques (six males; six females) housed at the German Primate Center. For management reasons, all animals were individually housed in indoor cages and were fed twice a day with commercial monkey chow supplemented with fruits and vegetables. Water was available *ad libitum*. Sample collection occurred between 06:00 and 07:30 AM after the lights were turned on in the housing room. For this, a plastic mat was placed underneath the cage of each animal and samples were collected upon urination. Only urine not contaminated with faeces was collected. Samples were brought to the Endocrinology Laboratory within two hours of collection where they were immediately processed for the different experimental treatments as outlined below.

Initially, three aliquots of 50 μL from each urine sample were immediately stored frozen to serve as controls. All experiments were conducted with the 12 samples collected, except for one experiment (storing samples at room temperature, see below) which was conducted on 11 samples due to insufficient urine volume available for one sample.

All experiments described were based on samples collected non-invasively (i.e. animals were not handled at all), conformed to the ABS/ASAB guidelines for the ethical treatment of animals, and were conducted in accordance with the recommendations of the Weatherall report on the use of non-human primates in research. They were also approved by the Ethics Committee of the German Primate Center.

### Urinary Neopterin And Creatinine Analysis

Urinary NEO concentrations were measured using a commercial Neopterin ELISA Kit from IBL International GmbH, Hamburg, Germany (Art. No. RE59321). The assay has been previously biologically validated with rhesus macaque urine^29^. Prior to assay, urine samples were diluted 1:10 to 1:200 with assay buffer (depending on the concentration of NEO in the sample) to bring the samples into the sensitivity range of the assay, and 20 μL of the diluted urine was then assayed using the manufacturer provided protocol. Minimum assay sensitivity was 0.34 ng mL^−1^. Inter-assay coefficients of variation, determined by repeated measurement of high and low value internal quality controls in each assay were 10.1% and 7.6%, respectively.

To adjust for differences in urine concentration, urinary NEO values were indexed to urinary creatinine (Cr) measured using the Jaffé reagent method^13^. As researchers using field samples would measure both Cr and urinary NEO concentrations from the same sample, we took the Cr value from the same sample as the urinary NEO concentration in each experimental treatment, rather than from controls. As such, our results show how indexed urinary NEO concentrations (those that would be of interest to field researchers) respond to the different treatments. Intra- and inter-assay coefficients of variation calculated from low and high value quality controls run in each assay were < 5%. Urinary NEO concentrations are presented as urinary NEO ng/mg Cr. As Cr is also widely used when indexing other urinary analytes such as steroid hormones or C-peptides^39^, we also report the effects of the different treatments on Cr concentrations (as mg/mL urine) separately.

### Collection And Storage Issues

#### Contamination With Soil And Faeces

Since urine samples collected in the wild may be contaminated with soil or faecal matter, we tested for a potential effect of this contamination on indexed urinary NEO concentrations. For this, 450 μL urine of each sample was pipetted into polypropylene tubes which contained 50 ± 5 mg of soil or freshly collected rhesus macaque faeces. Each contaminated sample was briefly vortexed and incubated for 5 min at room temperature before the supernatant was pipetted off into a clean tube. All samples were placed in the fridge (4°–6 °C) for 5 hours thereafter (to mimic storage in a cooler in the field) and subsequently stored at −20 °C until analysis.

#### Storage Of Samples At Room Temperature For Various Periods With And Without A Preservative

We tested the vulnerability of urinary NEO and Cr for a degradation effect by keeping urine samples at room temperature for a period of up to 3 weeks before freezing them. Specifically, urine samples were portioned into five aliquots of 100 μL each. While one set of samples was immediately stored frozen (control samples), all other samples were placed in the dark at ambient temperature (22–23 °C) for 2, 7, 10 and 21 days after which they were also placed at −20 °C. All samples were stored frozen until analysis together with the matched controls.

Since potential degradation of urinary analytes by microbial activity may be prevented by the addition of a biocide to the urine sample^36^, we repeated the storage experiment and tested the stability of urinary NEO and Cr concentrations in urine to which ProClin 200, a new generation antimicrobial agent, was added. For this, urine samples were portioned into five aliquots of 90 μL each to which 10 μL of a 1:30 watery dilution of ProClin 200 (Sigma-Aldrich Chemie, Taufkirchen, Germany) was added (working concentration of ProClin in sample: 0.33%). Again, one set of samples was immediately stored frozen (control samples), all other samples were placed in the dark at ambient temperature (22–23 °C) for 2, 7, 10 and 21 days after which they were also placed in the freezer. All samples were stored frozen until those from day 2, 10 and 21 were analysed together with the matched controls. Day 7 samples were not analysed since analysis of samples from day 2 and 10 revealed no difference in urinary NEO concentrations compared to controls (see Results).

Since urinary NEO and Cr concentrations in preserved samples were unaffected by storing samples for 3 weeks at ambient temperature (see Results), we repeated the storage experiment with ProClin 200, but now storing samples for 2 and 4 months at room temperature using urine that was left over from the contamination experiment. After the respective storage periods samples were diluted for Cr and urinary NEO measurements and dilutions were stored frozen until analysis together with matched frozen controls.

#### Collection Of Samples Onto Filter Paper

In order to assess the recovery of urinary NEO and Cr concentrations from urine stored on filter paper, we pipetted 200 μL of each urine sample on 2.5 cm squares of filter paper (Grade 903, Schleicher and Schuell, Dassel, Germany) following Higham *et al.*^22^ (which in turn was derived from Knott^41^). The filter paper squares were dried on aluminium foil placed on silica desiccant at room temperature (22–23 °C) and protected from light for 2 days. Thereafter, for measurement, we punched each filter paper square with a hand-held hole punch and removed five circles directly into a polypropylene test tube^22^. The punched circles were then eluted with 800 μL urinary NEO assay buffer by briefly vortexing each tube and placing the tubes in the fridge overnight. The next day, each sample was briefly vortexed again after which 2 × 300 μL were pipetted off and stored at −20 °C until analysis.

Following sample measurements, we corrected for the fact that we had only taken a portion of the filter paper square by calculating the surface area of all five circles, calculating what proportion of the whole 2.5 cm squares this represented, and therefore what proportion of the 200 μL original urine volume we had taken. We then corrected for this when calculating our urinary NEO and Cr concentrations^22^.

### Processing And Long-Term Storage Issues

#### Lyophilisation

Lyophilisation (freeze-drying) should produce samples that are extremely stable over time, and this process is often applied for stabilizing sensitive compounds in biological samples^22^,^42^. However, this method is only viable if good recovery of analyte concentrations following lyophilisation can be demonstrated^22^. We tested the recovery of urinary NEO and Cr after lyophilisation of urine, preparing aliquots of 150 μL of each urine sample in polypropylene cups. All aliquots were placed at −20 °C for 1 week. Samples were then freeze-dried overnight and stored frozen thereafter until analysis. Prior to analysis, 150 μL Millipore purified water was added to each lyophilized sample for analyte reconstitution. Samples were vortexed twice for 10 sec each and kept at room temperature for at least 30 min before diluting the sample for urinary NEO and Cr measurements.

#### Long-Term Freezing

In order to test the stability of urinary NEO and Cr levels after long-term storage of urine at sub-zero temperatures, we re-analysed urine samples (n = 12) that were measured for urinary NEO in a different study after 7-8 months of storage in a regular freezer (−20 °C).

#### 24-Hour Freeze-Thaw Cycles

In order to evaluate the effect of thawing and re-freezing and storing samples at ambient temperature for one day (as to simulate power loss or inconstant electricity supply of a freezer, or thawing during transport for analyses) on urinary NEO and Cr levels, we pipetted 250 μL from each urine sample into polypropylene cups and stored these frozen at −20 °C. After 3 days stored frozen, samples were thawed and stored in the dark at room temperature for 24 hours. From each sample 75 μL was pipetted off and stored at −20 °C together with the remaining urine (1^st^ freeze thaw cycle). The procedure was repeated 4 and 7 days later to provide samples that had been thawed and refrozen twice or three times, respectively (2^nd^ and 3^rd^ freeze/thaw cycle). Samples were finally stored frozen at −20 °C until analysis.

#### Data Analysis

As sample sizes were small, we used non-parametric statistics. We used the Wilcoxon signed rank test to assess the effects of a single treatment, and Friedman tests for experiments containing repeated-measures over time. In these latter cases, we undertook post-hoc paired exact Wilcoxon signed rank tests to determine the period after which effects on values over time first became significant. For all treatments, we undertook Spearman correlations to investigate whether treated and control values were correlated irrespective of whether the treatment resulted in a significant change in urinary NEO and Cr concentrations or not. All statistics were undertaken in R 2.7.0. Two-tailed tests using exact probabilities were performed, with analyses considered significant when P < 0.05.

## Additional Information

**How to cite this article**: Heistermann, M. and Higham, J. P. Urinary neopterin, a non-invasive marker of mammalian cellular immune activation, is highly stable under field conditions. *Sci. Rep.* **5**, 16308; doi: 10.1038/srep16308 (2015).

## Figures and Tables

**Table 1:** Summary of urinary NEO (ng/mg Cr) results.

TreatmentGroup	Treatment	Values as %of control(mean ± s.e.m.)	Friedman test		Wilcoxon test		Spearmancorrelationagainst control	
			χ^2^	P	T^+^	P	r_s_	P
Collection and medium-term storage	Contamination with soil	96.1 ± 3.6			18	0.206	0.965	**<0.0001**
	Contamination with faeces	98.3 ± 4.2			18	0.204	0.932	**<0.0001**
	Collection onto filter paper	110.4 ± 4.1			64	0.052	0.951	**<0.0001**
	21 days storage at RT without preservative	157.1 ± 9.3	27.839	**<0.001**			0.891	**<0.001**
	21 days storage at RT with ProClin 200	103.0 ± 4.6	1.403	0.705			0.964	**<0.0001**
Processing and long-term storage	2 months storage at RT with ProClin 200	114.3 ± 3.4			78	**<0.001**	0.965	**<0.0001**
	4 months storage at RT with ProClin 200	129.7 ± 3.6			78	**<0.001**	0.923	**<0.0001**
	Lyophilisation	100.6 ± 2.0			44	0.718	0.993	**<0.0001**
	8-months storage in freezer	105.4 ± 3.9			27	1.000	0.974	**<0.0001**
	Three 24-hour freeze-thaw cycles	101.6 ± 4.1	8.667	**0.013**			0.986	**<0.0001**

RT = ambient temperature (22−23 °C); values in bold indicate statistical significance.
